# MIMO Self-Heterodyne OFDM Using Band Selection Technique

**DOI:** 10.3390/e23010032

**Published:** 2020-12-28

**Authors:** Amira I. Zaki, Mai Abdelgelil, Said E. El-Khamy, Waleed K. Badawi

**Affiliations:** 1 Electronics and Communication Engineering Department, Arab Academy for Science, Technology and Maritime Transport (AASTMT), Alexandria 1029, Egypt; amzak10@aast.edu (A.I.Z.); mai.abdelgelil15@gmail.com (M.A.); elkhamy@ieee.org (S.E.E.-K.); 2 Electrical Engineering Department, Faculty of Engineering, Alexandria University, Alexandria 21544, Egypt

**Keywords:** self-heterodyne, OFDM, space time coded block, band selection frequency space time block coded (BS-FSTBC), space time block coded approach (STBC), band selection space time block coded (BS-STBC)

## Abstract

The 5G technology is a promising technology to cope with the increasing demand for higher data rate and quality of service. In this paper, two proposed techniques are implemented for multiple input multiple output (MIMO) self-heterodyne OFDM system to enhance data rate and minimize the bit error rate (BER). In both of the two proposed techniques, Band Selection (BS) approach is used, once with Space Time Block Coded (STBC) for the first proposed technique (BS- STBC), and once again with Frequency Space Time Block Coded (FSTBC) for the second proposed technique (BS-FSTBC). The use of the BS in the proposed techniques helps to choose the sub-band with better subchannels gains for sending the information and consequently, minimize the BER. Moreover, the use of the FSTBC instead of STBC helps to use the spectral efficiently and hence increase data rate. The simulation results show that the proposed techniques BS-STBC and BS-FSTBC, for the MIMO self-heterodyne OFDM system, provide a great enhancement in the BER performance when compared to the conventional techniques. Moreover, the simulation results show that the first proposed technique BS-FSTBC outperform the second propose technique BS-STBC in term of the BER performance.

## 1. Introduction

Due to the rapid increase in the number of users and the need for higher data rates, free bands and more bandwidth are required in the new 5G technology. This attracted the researchers′ attention to utilizing the millimeter-wave (mm-wave) bands in 5G [1]. The mm-wave communication is characterized by the high data rate as it occupies wide bandwidth at the high-frequency range (30 GHz–300 GHz) [2,3].

Despite the advantages of using the mm-wave band in 5G communication [4], however, it is difficult to design an oscillator for the mm-wave band with the same frequency stability of the UHF or VHF bands [5]. Moreover, the radio frequency (RF) components will create phase noise at the receiver and hence, techniques to handle these issues are required [6]. In order to manage these issues, the researchers in [7] and [8] suggested utilizing the self-heterodyne receiver in mm-wave communication. At the transmitter side of the self-heterodyne system, the modulated signals along with the local carrier are transmitted through the mm-wave band for the up-conversion. At the receiver side, the down-conversion is done to estimate the transmitted symbols using a square-law device, in which, the received local carrier is mixed with the modulated signals to get the estimated symbols [9]. Hence, by using a self-heterodyne receiver, the problem of phase noise and frequency offset are evaded. Moreover, at the self-heterodyne receiver, no local oscillator or phased locked loop (PLL) is required. Therefore, the system is more stable and has a lower cost and complexity of the transmitter [9].

In [9], a single-input-single-output (SISO) self-heterodyne orthogonal frequency division multiplexing (OFDM) system is proposed. The data symbols in [9] are transmitted on half of the available bandwidth, while the carrier frequency is transmitted on the other half, leaving a guard band between OFDM subcarriers and the RF carrier. Although the guard band is necessary to eliminate the interference caused by the nonlinear device, however, it diminishes the efficiency of bandwidth to the half. Based on this, researchers in [10,11] used the suggested receiver in [9] to replace the conventional super-heterodyne receiver.

The space time block code (STBC) is widely used by researchers in various research area with different systems and techniques, such as multiple input multiple output (MIMO) system [12,13,14], Orthogonal Transform Division Multiplexing (OTDM) technique [15], Generalized Spatial Modulation (GSM) and antenna grouping system [16], dual-hop full-duplex relay networks [17], visible light communications system [18], and**** heterogeneous system [19]. The used of the STBC eliminates the multipath effects and hence, enhance the performance of these systems in terms of bit error rate.

In [12], a STBC MIMO self-het OFDM system is considered in which, two transmitting antennas and two receiving antennas are used. Moreover, the STBC is used in order to remove the multipath effects and improve stability of the system. In this system, Alamouti code [20,21] was used with MIMO self-het OFDM, and the results show that the system performance is decreased when compared to the conventional MIMO STBC OFDM system. This degradation in system performance occurs due to the decrease in bandwidth efficiency, by transmitting the symbols on half of the bandwidth [12]. Moreover, the proposed system in [12] solved the problem of the phase noise at the receiver and the receiver complexity at the expense of the system BER performance. In [22], FSTBC is used with MIMO self-het OFDM system. The authors in [22] try to solve the problem of the bandwidth efficiency degradation that found in [12] by using the same technique but for four transmitting antennas and transmitting the symbols on the whole bandwidth. The symbols are transmitted alternatively on different half sub bands. In [22], four symbols are transmitted in single time slot. While in [12] only two symbols are transmitted per time slot. Thus, the technique in [22] uses the bandwidth more efficiently and hence increases the data rate compared with technique in [12]. However, the technique in [22] increases data rate and enhance BER performance but at the expense of the computational complexity.

The authors in [12,22] used STBC and FSTBC, respectively, for a MIMO self-het OFDM system. However, the proposed techniques in our paper utilize the band selection (BS) approach with STBC and FSTBC for MIMO self-het OFDM system. In this paper, two techniques are proposed to enhance the performance for MIMO self-het OFDM system in terms of data rate and BER. The main idea of the proposed techniques is to use the BS approach once with STBC [12] for the first proposed technique, BS-STBC, and once again with FSTBC [22] for the second proposed technique, BS-FSTBC. The use of the BS approach with STBC and FSTBC techniques help to select the optimum channel band to transmit the data over the MIMO channel and avoid the channel fading effects. The used of the optimum sub band with higher gain for transmission reduces the probability of error and consequently improve the BER performance. Moreover, maximum likelihood combiner is used at the receiver to separate the transmitted symbols for the proposed techniques. The simulation results show that the proposed techniques BS-STBC and BS-FSTBC for the MIMO self-heterodyne OFDM system, have superiority over the conventional techniques [12,22] in terms of BER performance. Hence, by using the BS technique, the performance of the STBC [12] and FSTBC [22] techniques are improved in term of BER. Moreover, the simulation results show that the first proposed technique BS-FSTBC outperform the second proposed technique BS-STBC in term of BER performance.

The rest of this paper is organized as follows; in Section 2, the system model of the (FSTBC) MIMO self-het OFDM system is presented. Section 3 presents the BS proposed technique in the STBC MIMO self-het OFDM system and the FSTBC MIMO self-het OFDM system. Section 4 presents and discuss the simulation results of the proposed approaches. Finally, the paper is concluded in Section 5.

Throughout this paper, the following notation is adopted: matrices are denoted by boldface capital letters, column vectors are represented by boldface lowercase letters, symbols are denoted in italics, $H_{ra}^{\;}$ is the channel gain between the *a*th transmitting antenna and *r*th receiving antennas,$\;\alpha_{c}^{\;}$ and,$\;\beta_{c}$ are the sum of channel gain to the 1st and 2nd receiving antenna respectively, ℛ{·} represents the real component of a complex number, (*) is the conjugate operation,$\;{\left(\cdot \right)}^{-1}$ inverse operation, ${\left(\cdot \right)}^{†}\;$ the pseudo-inverse operation, ${\left(\cdot \right)}^{H}$ is the hermition matrix, |·| is absolute operator.

## 2. The FSTBC MIMO Self-Het OFDM System

**Figure 1:**
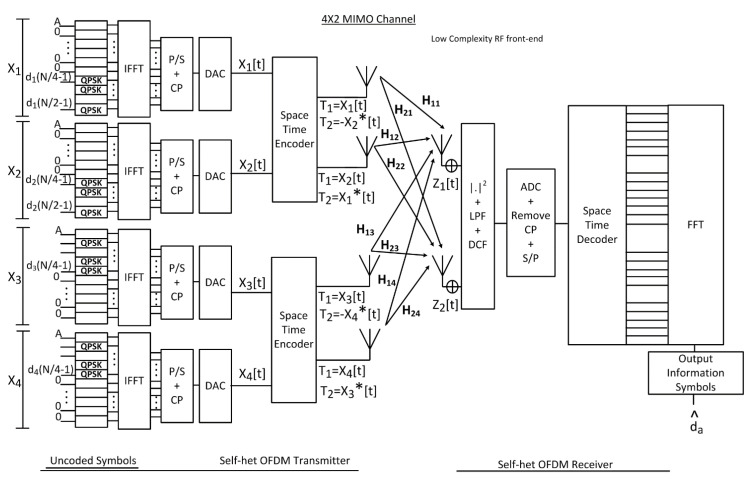
FSTBC self-het OFDM Proposed System Model.

The system model of the FSTBC MIMO self-het OFDM consists of four antennas at the transmitter and two antennas at the receiver as shown in Figure 1. The H $\in C^{A_{t\;}\times A_{r\;}}$ is channel response, where $A_{t}$ is the number of transmitting antennas and $A_{r}$ is the number of receiving antennas. The information subcarriers are divided into $A_{s}$ and $A_{g}$ which represent the OFDM number of subcarriers used to transmit the information symbols and the number of subcarriers that are zero-padded in each self-het OFDM transmitter respectively.

It is shown in Figure 1, that the proposed system transmits four OFDM symbols on two successive symbol intervals using four transmitting antennas and two receiving antennas. The proposed system increases the data rate to double that proposed by the conventional STBC MIMO self-het OFDM system. This is in addition to the increase in the diversity gain due to the increase in the number of transmitting antennas. The proposed system performs multiplexing in the spatial time and frequency domain, where the first and second symbol intervals are space-time encoded in the lower band of sub-carriers (zeros are embedded in the upper band), while the third and fourth symbols are space-time encoded in the upper band (zeros are embedded in the lower band). Thus, each pair of successive symbols can be received and separated at the receiver depending on the sub-band they were sent on (i.e., frequency multiplexing on two sub-bands).

The transmitted data symbols X are modulated using QPSK modulation. Then the modulated data symbols are divided into four code matrices. These code matrices are transmitted by $A_{t}$ transmitting antennas over $A_{s}$ sub-carriers. The code matrices are successively defined as:

(1) $$d_{1}[L]\;=\;X\left[1+4\left(L-1\right)\right],\;\mathrm{where}\;L=\;1,2,\;\ldots ,\;\frac{N}{4}.$$

(2) $$d_{2}[L]\;=\;X\left[2+4\left(L-1\right)\right],\;\mathrm{where}\;L=\;1,2,\;\ldots ,\;\frac{N}{4}.$$

(3) $$d_{3}[L]\;=\;X\left[3+4\left(L-1\right)\right],\;\mathrm{where}\;L=\;1,2,\;\ldots ,\;\frac{N}{4}.$$

(4) $$d_{4}[L]\;=\;X\left[4+4\left(L-1\right)\right],\;\mathrm{where}\;L=\;1,2,\;\ldots ,\;\frac{N}{4}.$$

where *N* is the number of the transmitted data symbols and $d_{a}$ is code matrices for each transmitter antenna (*a* = 1, 2, …, A_t_) [22]. The modulated data symbols are applied to the inverse fast Fourier transform (IFFT) stage. The IFFT input vectors for each transmitter antenna (*a*) are represented as (size ($1\times \frac{N}{2})$:

(5) $$X_{a}\left[K\right]=\{\begin{array}{cc} A & \;K=0\\ 0 & \;\;\;\;K=1,\cdots ,\frac{N}{4}-2\\ d_{a} & \;\;\;\;\;\;K=\frac{N}{4}-1,\cdots ,\frac{N}{2}-1 \end{array},\;a\;=\;1,\;2$$

(6) $$X_{a}\left[K\right]=\{\begin{array}{cc} A & \;\;\;K=0\\ d_{a} & \;\;\;K=1,\cdots ,\frac{N}{4}\\ 0 & \;\;\;\;\;\;\;\;K=\frac{N}{4}+1,\cdots ,\frac{N}{2}-1 \end{array}\;a\;=\;3,\;4,$$

where *K* =$\;\frac{N}{2},$ is the number of subcarriers and also the size of IFFT/FFT (*K =* $A_{g}$*+*$A_{s}$). The OFDM signal in the time domain $x_{a}^{\;}\left(t\right)$ is generated as:

(7) $$x_{a}\left(t\right)=ℛ\;\left\{\overset{\frac{N}{2}\;-1}{\underset{K=0}{\sum }}{\;X}_{a}\left[K\right]{\;e}^{j2\pi \;\Delta \zeta \left(\;K\;\right)t}\;\;\;\;\right\},a=1,\cdots ,\;4,$$

where Δζ is OFDM subcarrier frequency spacing [22]. Then, the parallel-to-series (P/S) conversion is applied to the output of the IFFT stage and after that the cyclic prefix (CP) is added to eliminate the inter symbol interference (ISI). A digital-to-analogue-converter (DAC) is then used to remove the high frequencies. Then, the transmitter encodes the information signal by the space time encoder.

Let $x=\left[x_{1},x_{2},x_{3},x_{4}\right]$ be the information symbols vector, in time domain, which is transmitted on each of the OFDM subcarrier, then the code-word matrix which is generated by Alamouti code is represented in a matrix form as:

(8) $$X_{c}=\left[\begin{array}{c} \begin{array}{cc} x_{1}^{\;} & \;-\;x_{2}^{*}\\ x_{2}^{\;} & x_{1}^{*}\\ x_{3}^{\;} & -\;x_{4}^{*}\\ x_{4}^{\;} & x_{3}^{*} \end{array} \end{array}\right].$$

Let $\alpha_{c}^{\;}≜{\;H}_{11}+H_{12}+H_{13}+H_{14}\;$ be the sum channel gains to the 1st receiving antenna from the four transmitting antennas and $\beta_{c}≜{\;H}_{21}+H_{22}+H_{23}+H_{24}$ is the sum of channel gains of the 2nd receiving antenna from the four transmitting antennas. Then, the received signal at each receiving antenna is given by:

(9) $$\;Y\;=\;H_{\mathrm{eq}\;}x+Z,$$

where Z is the AWGN components and $H_{\mathrm{eq}\;}$ is the channel MIMO which represented in a matrix form as:

(10) $$H_{\mathrm{eq}}\;≜\;\left[\begin{array}{llll} \alpha_{c}^{*}H_{11} & \alpha_{c}^{*}H_{12} & \alpha_{c}^{*}H_{13} & \alpha_{c}^{*}H_{14}\\ \beta_{c}^{*}H_{21} & \beta_{c}^{*}H_{22} & \beta_{c}^{*}H_{23} & \beta_{c}^{*}H_{24}\\ \alpha_{c}H_{12}^{*} & -\alpha_{c}H_{11}^{*} & \alpha_{c}H_{14}^{*} & -\alpha_{c}H_{13}^{*}\\ \beta_{c}H_{22}^{*} & -\beta_{c}H_{21}^{*} & \beta_{c}H_{24}^{*} & -\beta_{c}H_{23}^{*} \end{array}\right],$$

where $H_{ra}$ is the channel gain between the *a*th transmitting antenna and *r*th receiving antennas and (*) is the conjugate operation. The channel is assumed to be constant during two successive symbol intervals [22].

At the receiver side, no local carrier oscillator or recovering of carrier phase is required. A low pass filter (LPF) with a cut-off frequency equal to $f_{g}\;$+$\;f_{s}$ and filters of DC components are used to eliminate the high frequency signals and the DC components. Then, the CP is removed, and the fast Fourier transform (FFT) is performed after serial-to-parallel (S/P) conversion. Thus, the received signal can be expanded as:

(11) $$[\begin{array}{l} y_{11}\\ y_{21}\\ y_{12}^{*}\\ y_{22}^{*} \end{array}]\;=\;[\begin{array}{llll} \alpha_{c}^{*}H_{11} & \alpha_{c}^{*}H_{12} & \alpha_{c}^{*}H_{13} & \alpha_{c}^{*}H_{14}\\ \beta_{c}^{*}H_{21} & \beta_{c}^{*}H_{22} & \beta_{c}^{*}H_{23} & \beta_{c}^{*}H_{24}\\ \alpha_{c}H_{12}^{*} & -\alpha_{c}H_{11}^{*} & \alpha_{c}H_{14}^{*} & -\alpha_{c}H_{13}^{*}\\ \beta_{c}H_{22}^{*} & -\beta_{c}H_{21}^{*} & \beta_{c}H_{24}^{*} & -\beta_{c}H_{23}^{*} \end{array}]\;[\begin{array}{l} x_{1}\\ x_{2}\\ x_{3}\\ x_{4} \end{array}]\;+\;[\begin{array}{l} z_{11}\\ z_{21}\\ z_{12}^{*}\\ z_{22}^{*} \end{array}],$$

where $y_{11}$ and $y_{21}$ are the received signals, in time domain, at the 1st receiving antenna during the 1st time slot and the second time slot successively. $y_{21}^{\;}$ and $y_{22}^{\;}$ are the received signals at the 2nd receiving antennas during the 1st and 2nd time slots respectively. As well, $z_{11}^{\;}$ and $z_{21}^{\;}$ are the AWGN noise components at the 1st receiving antenna during the 1st time slot and the second time slot successively. $z_{21}^{\;}$ and $z_{22}^{\;}$ are the AWGN noise components at the 2nd receiving antennas during the 1st and 2nd time slots respectively [22].

To apply the ZF equalizer, let $H_{\mathrm{eq}}^{†}\;$ be the pseudo-inverse of $H_{\mathrm{eq}}$ which is calculated and defined as follows:

(12) $$H_{\mathrm{eq}}^{†}\;≜\;{\left(H_{\mathrm{eq}}\;H_{\mathrm{eq}}^{H}\right)}^{-1}H_{\mathrm{eq}}^{H}=\frac{1}{C_{i}}\;H_{\mathrm{eq}}^{H},\;$$

where i = 1, 2 and the constants $C_{1}$ and $C_{2}$ are defined as:

(13) $${\;C}_{1}={\left|\alpha_{c}\right|}^{2}\left(\overset{2}{\underset{a=1}{\sum }}{\left|H_{1a}^{\;}\right|}^{2}\;\right)+\left|\beta_{c}\right|{\;}^{2}\left(\overset{2}{\underset{a=1}{\sum }}{\left|H_{2a}^{\;}\right|}^{2}\;\right),$$

(14) $${\;C}_{2}={\left|\alpha_{c}\right|}^{2}\left(\overset{4}{\underset{a=3}{\sum }}{\left|H_{1a}^{\;}\right|}^{2}\;\right)+\left|\beta_{c}\right|{\;}^{2}\left(\overset{4}{\underset{a=3}{\sum }}{\left|H_{2a}^{\;}\right|}^{2}\;\right),$$

where *a* is *a*th transmit antenna and $H_{\mathrm{eq}}^{H}$ is the hermition of $H_{\mathrm{eq}}$ which can be represented in a matrix form as:

(15) $$H_{\mathrm{eq}}^{H}\;≜\;\left[\begin{array}{llll} \alpha_{c}H_{11}^{*} & \beta_{c}H_{21}^{*} & \alpha_{c}^{*}H_{12} & \beta_{c}^{*}H_{22}\\ \alpha_{c}H_{12}^{*} & \beta_{c}H_{22}^{*} & -\alpha_{c}^{*}H_{11} & -\beta_{c}^{*}H_{21}\\ \alpha_{c}H_{13}^{*} & \beta_{c}H_{23}^{*} & \alpha_{c}^{*}H_{14} & \beta_{c}^{*}H_{24}\\ \alpha_{c}H_{14}^{*} & \beta_{c}H_{24}^{*} & -\alpha_{c}^{*}H_{13} & -\beta_{c}^{*}H_{23} \end{array}\right].$$

At the receiving side, ZF equalizer and maximum likelihood detection (MLD) is utilized for recovering the symbols of information by consider the $\hat{x}=H_{\mathrm{eq}}^{†}Y=\;\frac{H_{\mathrm{eq}}^{H}}{C_{i}}\;Y\;$ vector. So, in order to detect the first transmitted symbol ${\hat{x}}_{1}^{\;}$, the first row in ${\;H}_{\mathrm{eq}}^{H}$ matrix in (15) is multiply by the received signal vector (**y**) in (11) to get resultant ${\hat{r}}_{1}$ symbol as [22]:

(16) $${\hat{r}}_{1}={\left|\alpha_{c}^{\;}\right|}^{2}{\left|H_{11}^{\;}\right|}^{2}x_{1}+{\left|\alpha_{c}^{\;}\right|}^{2}H_{11}^{*}H_{12}^{\;}x_{2}+{\left|\alpha_{c}^{\;}\right|}^{2}H_{11}^{*}H_{13}^{\;}x_{3}+{\left|\alpha_{c}^{\;}\right|}^{2}H_{11}^{*}H_{14}^{\;}x_{4}+\;{\left|\beta_{c}^{\;}\right|}^{2}{\left|H_{21}^{\;}\right|}^{2}x_{1}+{\left|\beta_{c}^{\;}\right|}^{2}H_{21}^{*}H_{22}^{\;}x_{2}+{\left|\beta_{c}^{\;}\right|}^{2}H_{21}^{*}H_{23}^{\;}x_{3}+{\left|\beta_{c}^{\;}\right|}^{2}H_{21}^{*}H_{24}^{\;}x_{4}+\;{\left|\alpha_{c}^{\;}\right|}^{2}{\left|H_{12}^{\;}\right|}^{2}x_{1}-{\left|\alpha_{c}^{\;}\right|}^{2}H_{11}^{*}H_{12}^{\;}x_{2}+{\left|\alpha_{c}^{\;}\right|}^{2}H_{14}^{*}H_{12}^{\;}x_{3}-{\left|\alpha_{c}^{\;}\right|}^{2}H_{13}^{*}H_{12}^{\;}x_{4}+\;{\left|\beta_{c}^{\;}\right|}^{2}{\left|H_{22}^{\;}\right|}^{2}x_{1}-{\left|\beta_{c}^{\;}\right|}^{2}H_{21}^{*}H_{22}^{\;}x_{2}+{\left|\beta_{c}^{\;}\right|}^{2}H_{24}^{*}H_{22}^{\;}x_{3}-{\left|\beta_{c}^{\;}\right|}^{2}{\;H}_{23}^{*}H_{22}^{\;}x_{4}+\;\;z_{22}^{*}\;+{\;z}_{11}^{\;}+{\;z}_{21}^{\;}+{\;z}_{12}^{*}.$$

As seen in Equation (16) the coefficients of the second transmitted symbol $x_{2}$ are in opposite polarity and hence, they cancel each other. The terms of $x_{3}\;\mathrm{and}\;x_{4\;}$ are discarded (filtered out) because they are transmitted on the other sub-band (the upper band). By simplifying, (16) can be calculated as:

(17) $${\hat{r}}_{1}=\left[{\left|\alpha_{c}^{\;}\right|}^{2}{\left|H_{11}^{\;}\right|}^{2}+{\left|\beta_{c}^{\;}\right|}^{2}{\left|H_{21}^{\;}\right|}^{2}+{\left|\alpha_{c}^{\;}\right|}^{2}{\left|H_{12}^{\;}\right|}^{2}\;+{\left|\beta_{c}^{\;}\right|}^{2}{\left|H_{22}^{\;}\right|}^{2}\right]{\;x}_{1}+\mathrm{AWGN}\;\mathrm{components}.$$

Then the result in Equation (17) is divided by $C_{1}$ to estimate first the transmitted symbol ${\hat{x}}_{1}^{\;}$. However, in case of detecting ${\hat{x}}_{2}^{\;}$, the second row in $H_{\mathrm{eq}}^{H}$ matrix is used. Similarly, for detecting the third and the fourth transmitted symbols (${\hat{x}}_{3}^{\;}$ and ${\hat{x}}_{4}^{\;}$), the third and the fourth rows in $\;H_{\mathrm{eq}}^{H}$ matrix are used respectively. However, in case of detecting ${\hat{x}}_{3}^{\;}$ and ${\hat{x}}_{4}^{\;}$, the resultant for each of them is divided by $C_{2}$ and the term of $x_{1}\;\mathrm{and}\;x_{2}$ are discarded (filtered out) due to their existence on the other sub-band (the lower band).

## 3. Band Selection Proposed Technique

### 3.1. BS-STBC MIMO Self-Het OFDM System

The STBC MIMO self-het OFDM system increased the data rate compared by self-het OFDM system. The STBC MIMO self-het OFDM System achieves only half data rate due to the presence of the guard band. In the BS technique, to each transmitting antenna, the information will be sent according to the highest sub-channel gain in an upper half band or lower half band of the *K* subcarriers band. BS technique is proposed to avoid the channel fading effects in order to maximize the diversity gain and data rate.

To choose the highest sub-channel gain,${\;H}_{\max1}$ and $H_{\max2}$ are calculated as:

(18) $${\;H}_{\max1}=\arg\max\left(\;\overset{2}{\underset{a=1}{\sum }}\;\left|H_{1a}\right|{\;}^{2}\;\right),$$

(19) $${\;H}_{\max2}=\arg\max\left(\;\overset{2}{\underset{a=1}{\sum }}\;\left|H_{2a}\right|{\;}^{2}\;\right),$$

where $H_{\max1}$ and $H_{\max2}$ represent the sum of channel gains from two transmitting antennas to the 1st received antenna, the sum of channel gains from two transmitting antennas to the 2nd received antenna respectively and a is *a*th transmitting antenna.

**Figure 2:**
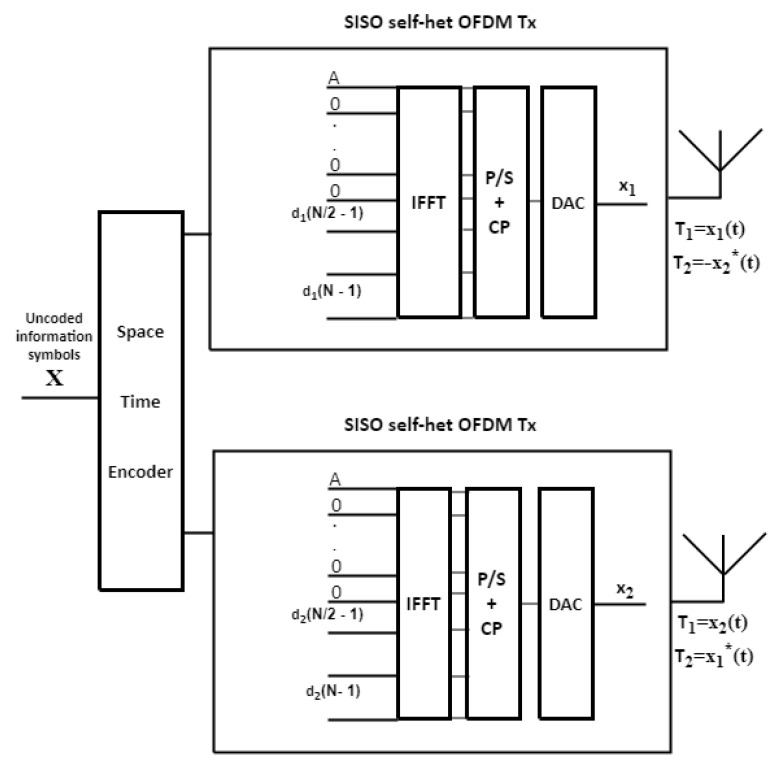
STBC MIMO self-het OFDM transmitter when the data is transmitted in the upper band.

**Figure 3:**
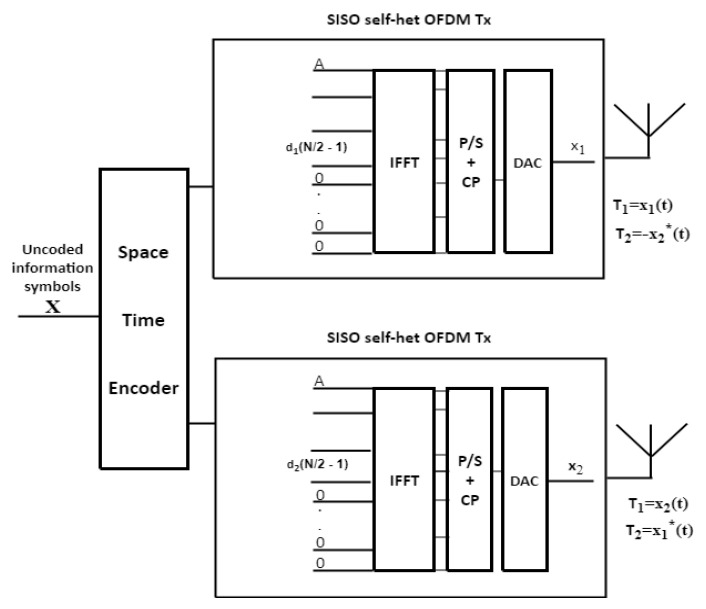
STBC MIMO self-het OFDM transmitter when the data is transmitted in the lower band.

Let i be the number of the $H_{\max1}$ multipath component and *q* is the number of the $H_{\max2}$ multipath component, then, there are three scenarios for performing the BS which are:

1. If the value of *i* and the value of *q* are in the upper band, the data symbols will be sent at the upper band in the two transmitting antennas as shown in Figure 2.
2. If the value of *i* and the value of *q* are in the lower band, the data symbols will be sent at the lower band in the two transmitting antennas as shown in Figure 3.
3. If the values of *i* and *q* are in different bands, the data will be sent through the band depending on the maximum value of H_max1_ and H_max2_.

A pilot bit = 1 is added to indicate the band which the data symbol will be sent on. At the receiver, if the bit = 1, the data symbols are sent on the upper band and if the bit ≠ 1, the data symbols are sent on the lower band.

### 3.2. BS-FSTBC MIMO Self-Het OFDM System

When the BS proposed technique is applied to the FSTBC MIMO self-het OFDM proposed system, the information symbols are sent according to the better sub-channel gains in upper band or lower band of the four transmitting antennas. To choose the highest sub-channel gain,${\;H}_{\max1}$ and $H_{\max2}$ are calculated as:

(20) $${\;H}_{\max1}=\left(\overset{2}{\underset{a=1}{\sum }}\left|H_{1a}\right|{\;}^{2}\right)+\left(\overset{2}{\underset{a=1}{\sum }}\left|H_{2a}\right|{\;}^{2}\right),$$

(21) $${\;H}_{\max2}=\left(\overset{4}{\underset{a=3}{\sum }}\left|H_{1a}\right|{\;}^{2}\right)+\left(\overset{4}{\underset{a=3}{\sum }}\left|H_{2a}\right|{\;}^{2}\right),$$

where $H_{\max1}$ represent the sum of channel gains from 1st and 2nd transmitting antennas to the 1st and 2nd receiving antennas, while ${\;H}_{\max2}$ is the sum of channel gains from 3rd and 4th transmitting antennas to the 1st and 2nd receiving antennas and a is *a*th transmitting antenna.

**Figure 4:**
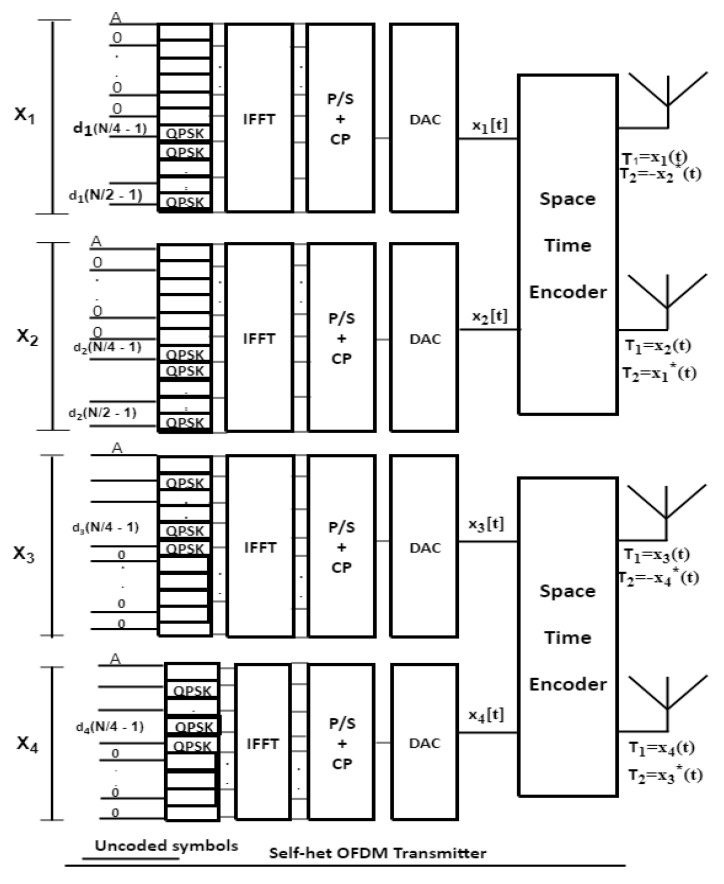
FSTBC MIMO self-het OFDM transmitter when the data is transmitted in the upper band for 1st and 2nd transmitting antennas and in the lower band for 3rd and 4th transmitting antennas.

**Figure 5:**
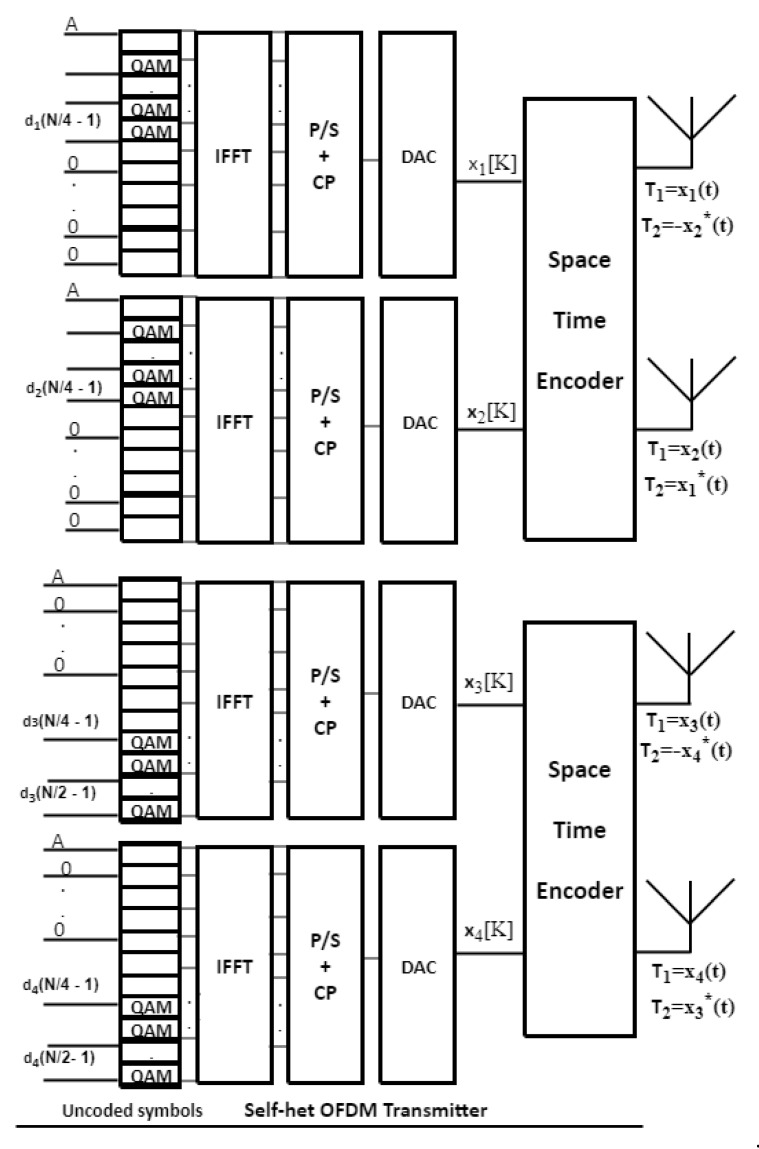
FSTBC MIMO self-het OFDM transmitter when the data is transmitted in the lower band for 1st and 2nd transmitting antennas and in the upper band for 3rd and 4th transmitting antennas.

Again, let *i* be the number of the $H_{\max1}$ multipath component and q is the number of the $H_{\max2}$ multipath component, then, there are three scenarios for performing the BS which are represented as follows:

1. If the value of *i* and the value of *q* are in the upper sub-band, the data will be sent on the upper sub-band in the 1st and 2nd transmitting antennas while the 3rd and 4th transmitting antennas will use the lower sub-band as shown in Figure 4.
2. If the value of *i* and the value of *q* are in the lower sub-band, the data will be sent at the lower sub-band on the 1st and 2nd transmitting antennas and the 3rd and 4th transmitting antennas will use the upper sub-band as shown in Figure 5.
3. If the value of *i* and the value of *q* are in different bands, the data will be sent on each pair of transmitting antennas through the band where the maximum values of H_max1_ and H_max2_ occure.

A pilot bit = 1 is added to indicate the band which the data symbol will be transferred on. At the receiver, if the bit = 1, the data symbols are sent on the upper sub-band for the 1st pair of transmitting antennas and on the lower sub-band for the 2nd pair of transmitting antennas and if the bit ≠ 1, the data symbols are sent on the lower sub-band for the 1st pair of transmitting antennas and on the upper sub-band for the 2nd pair transmitting antennas.

## 4. Simulation Results and Discussion

To evaluate the performance of the two proposed system models with band selection technique, a set of experiments has been conducted. These experiments examine Bit Error Rate (BER) performance of the proposed systems. Assume that a perfect and imperfect channel estimation (with 10% error over each symbol in each path delay for each channel). Moreover, the antenna channels are assumed to be uncorrelated. The simulation parameters of the experiments are summarized in Table 1, where OFDM with QPSK modulation is used.

**Figure 6:**
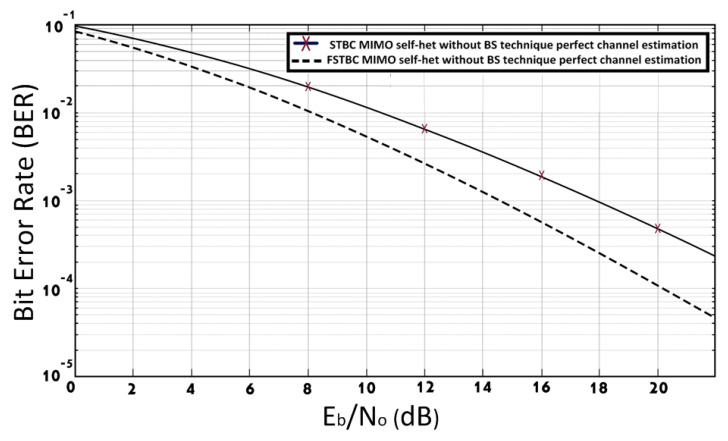
BER of FSTBC MIMO self-het OFDM and STBC MIMO self-het OFDM systems with perfect channel estimation.

Figure 6 shows the BER performance of the FSTBC MIMO self-het OFDM system and STBC MIMO self-het OFDM system without the use of the band section technique, assuming perfect channel estimation. As shown in Figure 6, the BER of $10^{-3}$ is achieved at SNR equal to 14.7 dB for FSTBC MIMO self-het OFDM system and 17.9 dB for STBC MIMO self-het OFDM system. Thus, the proposed system improves the BER performance by approximately 3 dB.

**Figure 7:**
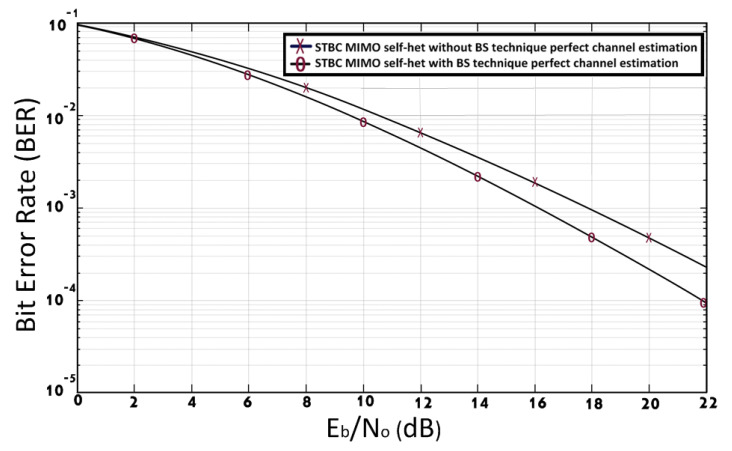
BER of STBC MIMO self-het OFDM system with and without [12] using BS technique, assuming perfect channel estimation.

Figure 7 compares the BER performance of STBC MIMO self-het OFDM system with and without using BS proposed technique, assuming perfect channel estimation. The simulation results in Figure 7 shows that, the STBC MIMO self-het OFDM system with BS technique improves the BER performance by 1.9 dB at BER of $10^{-3}$ when compared to the STBC MIMO self-het OFDM system without the BS technique [12]. The BER performance is improved due to the use of the BS approach in the proposed systems to select the optimum sub band for the transmission of each symbol over the different transmitted antenna. The used of optimum sub band with higher gain for transmission reduces the probability of error and consequently improve the BER performance. Thus, the capacity of the communication system will be increased by using the BS technique.

**Figure 8:**
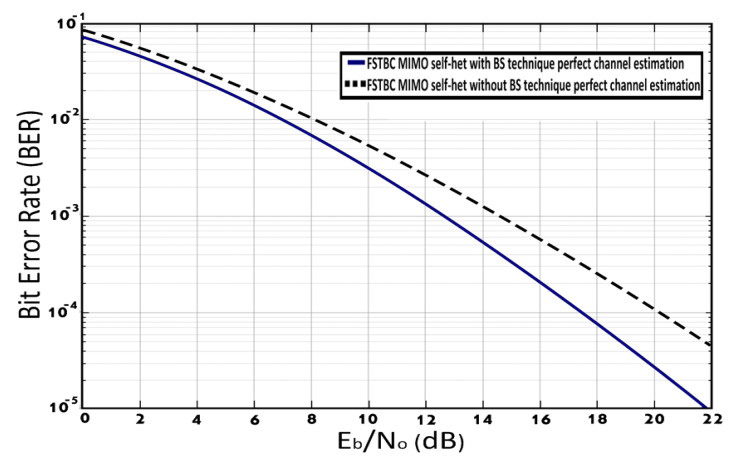
BER of FSTBC MIMO self-het OFDM system with and without [22] using BS technique assuming perfect channel estimation.

Figure 8 shows the BER performance of the FSTBC MIMO self-het OFDM system with and without [22] using BS technique, assuming perfect channel estimation. The simulation results in Figure 8 shows that the use of the BS technique with the FSTBC MIMO self-het OFDM system improves the BER performance by 2.1 dB at a BER of $10^{-3}$.

**Figure 9:**
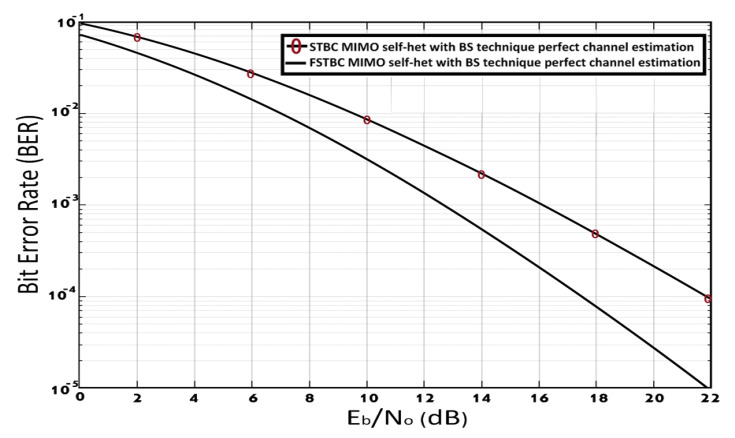
BER of FSTBC MIMO self-het OFDM system and STBC MIMO self-het OFDM system with BS technique, assuming perfect channel estimation.

**Figure 10:**
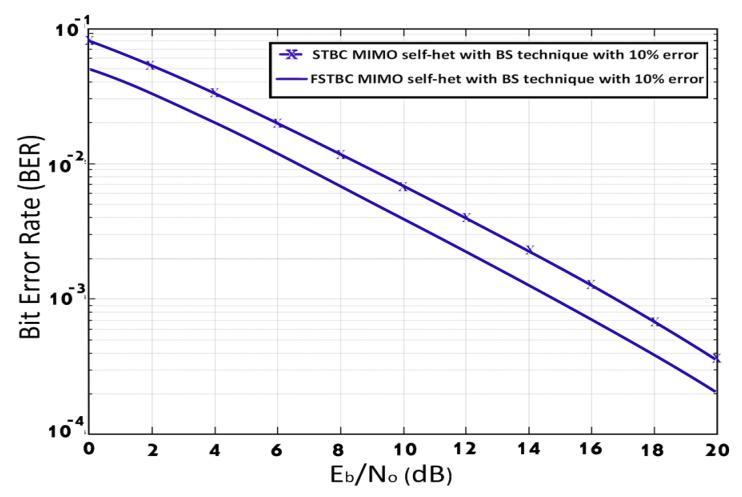
BER for STBC MIMO self-het OFDM system using BS technique and FSTBC MIMO self-het OFDM system using BS technique, assuming imperfect channel estimation.

Figure 9 illustrates the BER performance of the FSTBC MIMO self-het OFDM system with BS technique compared to STBC MIMO self-het OFDM system with BS technique, assuming perfect channel estimation. As shown in Figure 9, the FSTBC MIMO self-het OFDM system with BS technique obtains BER =$\;10^{-3}$ at SNR equal to 12.8 dB while the STBC MIMO self-het OFDM system with BS technique introduces the same BER at SNR equal to 16 dB. Therefore, FSTBC MIMO self-het OFDM system with BS technique outperform the STBC MIMO self-het OFDM system with BS technique by 3.2 dB. Figure 10 shows the BER performance of the STBC MIMO self-het OFDM system using BS technique compared to the FSTBC MIMO self-het OFDM system using BS technique, assuming imperfect channel estimation. As noticed in Figure 10, the FSTBC MIMO self-het OFDM system using BS technique improves the BER performance by 2 dB, at a BER of $10^{-3}$, when compared to the STBC MIMO self-het OFDM system using BS technique.

**Figure 11:**
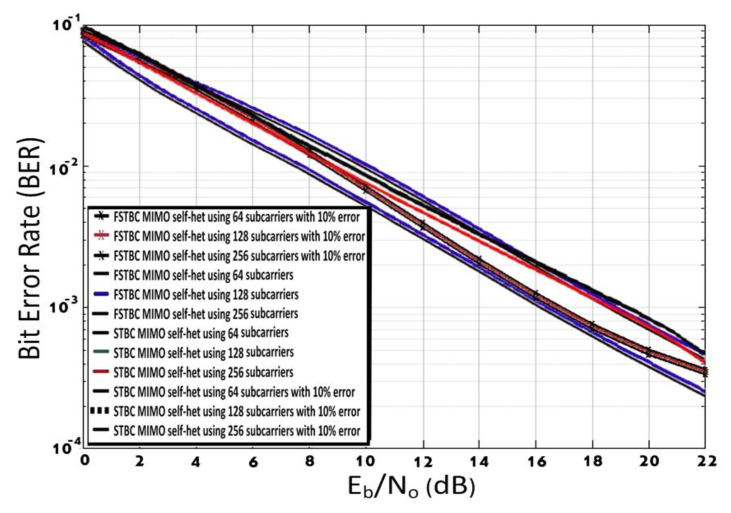
BER for STBC MIMO self-het OFDM system and FSTBC MIMO self-het OFDM system, assuming perfect and imperfect channel estimation for different numbers of subcarriers.

The performance of the STBC MIMO self-het OFDM system and FSTBC MIMO self-het OFDM system using different numbers of subcarriers (K = 64, 128, and 256), for perfect and imperfect channel estimation, is presented in Figure 11. The simulation results in Figure 11 show that, increase the number of the subcarrier has nearly no effect on the performance the FSTBC MIMO self-het OFDM system and STBC MIMO self-het OFDM system, for perfect and imperfect channel estimation. The performance is nearly the same and the curves are overlap. The FSTBC MIMO self-het OFDM system will have less complexity because it can introduce the desired BER performance with low number of subcarriers and less SNR compared to STBC MIMO self-het OFDM system. For example, FSTBC MIMO self-het OFDM system obtains the BER of $\;10^{-3}$ at a SNR equal to 17.0 dB, using 64 subcarriers, where imperfect channel estimation is assumed. While STBC MIMO self-het OFDM system obtains the BER of $\;10^{-3}$ at a SNR equal to 19.35 dB, using 64 subcarriers, where imperfect channel estimation is assumed. Hence, the FSTBC MIMO self-het OFDM system obtains the desired BER using a smaller number of sub carriers and lower SNR compared to STBC MIMO self-het OFDM system. Moreover, comparative details (SNR) for STBC MIMO self-het OFDM system and FSTBC MIMO self-het OFDM system, assuming perfect and imperfect channel estimation for different numbers of subcarriers at BER equal 10^−^^3^ are summarized in Table 2

## 5. Conclusions

In this paper, two new techniques; BS-STBC and BS-FSTBC are proposed for MIMO self-het OFDM system. The two proposed techniques are based on using BS approach. The use of BS helps to select the optimum sub band for data transmission. The used of the optimum sub band with higher gain for transmission reduces the probability of error and consequently improve the BER performance. The FSTBC achieves the full data rate compared to the STBC technique which achieves only the half data rate. The proposed techniques (BS-STBC), and (BS-FSTBC) are compared to the conventional techniques (STBC), and (FSTBC). The simulation results showed that proposed techniques outperform the conventional techniques performance in terms of BER. The proposed BS-FSTBC technique has superiority over the proposed BS-STBC technique in term of BER performance. The FSTBC MIMO self-het OFDM system can withstand the required same high BER performance level with a small number of subcarriers providing the lower complexity.

## Figures and Tables

**Table 1:** Simulation parameters [22].

Simulation Method	Monte Carlo	
Number of subcarriers (K)	512	
Number of transmitting antenna (A_t_)	STBC	FSTBC
	2	4
Number of receiving antennas (Ar)	2	
Sampling frequenc (f_s_)	1 MHZ	
Sampling time (T_s_)	10-6 s	
Modulation type	QPSK	
channel type	Rayleigh channel with four taps multipath components	

**Table 2:** SNR for STBC MIMO self-het OFDM system and FSTBC MIMO self-het OFDM system, assuming perfect and imperfect channel estimation for different numbers of subcarriers BER equal 10^−^^3^.

Channel Estimation	Techniques	SNR (dB) at 10^−3^ BER		
		Number of Subcarriers		
		64	128	256
**Perfect**	STBC MIMO self-het	18.5	19.0	18.53
	FSTBC MIMO self-het	16.7	16.4	16.2
**Imperfect** **(10% error)**	STBC MIMO self-het	19.35	19.33	19.36
	FSTBC MIMO self-het	17.0	17.06	16.9

## Data Availability

Not applicable.
